# Diabetic Patients' Perspective About New Technologies Used in Managing Diabetes Mellitus in Saudi Arabia: A Cross-Sectional Study

**DOI:** 10.7759/cureus.25038

**Published:** 2022-05-16

**Authors:** Omar M Al-Nozha, Esraa K Alshareef, Afnan F Aljawi, Enas T Alhabib, Raghad S AlMahweeti, Sarah A Aljuhani, Sawsan A Alamri, Ohoud S Alahmadi

**Affiliations:** 1 Internal Medicine, Taibah University, Madina, SAU; 2 Medicine and Surgery, Taibah University, Madina, SAU; 3 Medicine and Surgery, Taibah University, Medina, SAU

**Keywords:** diabetes mellitus, satisfaction, awareness, management, new technologies

## Abstract

Background: Diabetes technologies are hardware, devices, and software that are used by people with diabetes to manage their condition, from lifestyle interventions to the monitoring of blood glucose levels. The development of these technologies is advancing, but their use in Saudi Arabia is under-researched.

Objectives: To appraise the awareness of using new technological options in managing patients with diabetes and to assess the patients' satisfaction while using them.

Method: This was an e-questionnaire-based cross-sectional study. The targeted population of the study was patients with diabetes in Saudi Arabia. A total of 452 respondents participated in a survey in the period between 2020 and 2021. The collected data were analyzed using descriptive statistical methods and Chi-squared tests.

Results: Some 69% of participants were aware of the new technologies used in managing diabetes. There were discrepancies between the awareness and the use of new technologies. Several causes of non-use were identified; the main cause was high cost, as reported by more than half of non-users (53.2%). Other causes included non-availability and difficulty of use. Mobile health applications had the highest use rate (13.5%) among new technologies; patients reported using them mostly for blood glucose monitoring, physical activity, and nutritional programs. Patients' satisfaction was higher for modern technologies than for conventional methods.

Conclusion: The results indicate that awareness of the new technologies used in managing diabetes was higher than their use. Moreover, the use of modern technologies improved the satisfaction of patients.

## Introduction

Diabetes mellitus (DM) is one of the most common chronic diseases; around 537 million people aged 20-79 years were suffering from it at the end of 2021, and it is estimated that by 2030 the number will increase to be 643 million adults worldwide [1]. Recently the International Diabetes Federation (IDF) has reported the prevalence of DM in the Kingdom of Saudi Arabia (KSA) to be 18.3% among adults [1].

Diabetes technology is defined as “hardware, devices, and software that patients with diabetes use to manage their condition, from lifestyle to blood glucose levels” [2]. Recently, it has been divided into two main categories: insulin administered devices, starting from a simple insulin pen to advanced insulin pumps, and blood glucose monitoring devices which range from simple glucose meters to continuous glucose monitoring (CGM) devices [2]. Many studies have shown the efficacy of new technologies and an overall improvement in diabetes control and patient satisfaction. For CGM, hemoglobin A1c (HbA1c) reduction was shown in multiple studies [2-5] in both type 1 and type 2 DM and reductions in hypoglycemia specifically for type 1 patients. Insulin pumps were shown to reduce HbA1c and the frequency of hypoglycemia and hyperglycemia levels, especially when combined with sensors, and increased patients’ treatment satisfaction [2, 6]. A novel interstitial device known as flash glucose monitoring (FGM) which was used to obtain glucose levels instantly has been associated with improvements in HbA1c levels and reductions in hypoglycemia; it was also found to improve user satisfaction [7]. Another novel way to engage patients in their DM management is mobile health (‘mHealth’) which is a term describing digital health, defined by the WHO as “medical and public health practice supported by mobile devices” that are intended to improve health outcomes and quality of life by nutrition and exercise advice, encouraging glucose monitoring, the interpretation of results, adjusting medication doses, and decreasing complications [8]. Although health applications have been developing rapidly to help people manage their diabetes, the evidence of their effectiveness and safety for diabetes remains limited and requires evaluation of the accuracy, clinical validity, and quality [8].

Diabetes is a lifelong disease that requires continuous self-management in order to decrease the probability of long-term complications and prevent acute deterioration of the condition [9]. Better self-management leads to improvements in patients’ conditions, especially HbA1c, which is associated with a lower incidence of complications and a decrease in mortality rate among patients with diabetes [9]. Good education, using new technology, and follow-up can show significant improvements in the quality of life for people with diabetes [2].

Although these technologies are developing rapidly, the impact of their use in Saudi Arabia is still not clear. In our study, we aimed to explore patients' perspectives, awareness, and satisfaction regarding these new technologies.

## Materials and methods

Subjects

We used an e-questionnaire-based cross-sectional study in the period between 2020 and 2021, after obtaining the approval of the ethical and research committee of Taibah University, Madinah, Saudi Arabia. An electronic informed consent of each participant was obtained. The study has followed Helsinki Declaration in all stages. The target population was people with diabetes in the KSA. A sample size of 385 participants was calculated using the surveymonkey.com with a 5% margin of error and 95% confidence level. The actual sample size was 452 which was increased for better representation of the population. Patients with type 1, type 2, or gestational diabetes, aged between 18 and 60 years, who could read Arabic clearly and could use social media websites and applications, were included. There were no excluding criteria.

Data collection and research tool

The e-questionnaire was designed based on the literature review of new technologies used in diabetes management. It was distributed on social media websites with an electronic message to explain the purpose of our study. The questionnaire was divided into sections: the first part consisted of seven questions about demographic data, the second part consisted of health information, i.e., type of diabetes and HbA1c level, and then directed the participants to the following part of the questionnaire according to their answers. The last sections showed the different technologies and consisted of questions about patient awareness of these technologies (Appendix 1). The satisfaction section was inspired by a questionnaire used to assess treatment satisfaction of another study, with some changes to the items to adapt to the new technologies that we measured and the Likert scale was used to assess patient responses [10]. (see Table in Appendix 1).

The validity and reliability of the questionnaire were tested through a pilot study. The questionnaire was given in a face-to-face manner by the researchers to 38 patients with diabetes chosen randomly in an endocrine clinic. The pilot study sample was not included in the current study. Also, the questionnaire was reviewed by two health experts to explore whether any incomprehensible items led to misunderstandings. One of the problems we faced was that the patients could not differentiate between some of the technologies, so we added a brief explanation of each technology. Also, a section on glucometers was added. After the questionnaire reached its current final form, the poll was opened for two weeks and the data was collected automatically.

All questionnaire items were translated into the Arabic language by a healthcare physician and a translator expert fluent in both Arabic and English languages. The resulting Arabic questionnaire was then translated back into the English language by another two experts fluent in both languages. Those two experts were blinded to the questionnaire's original English version. The back-translated version of the questionnaire was compared with the original English one to check the translation quality which is the back-translation method recommended by the World Health Organization (WHO) [11].

Data analysis

The statistical analysis was done using Statistical Package for Social Sciences (SPSS) software, version 22.0, for Windows (SPSS, Inc., Chicago, IL). The demographic- and diabetes-related data of the 452 patients with diabetes studied were tabulated and presented in frequency number, percentage, mean, and standard deviation. The awareness of the patients of the new technologies in managing diabetes was calculated. The frequency of use of different new health technologies among the studied patients was tabulated. Satisfaction was calculated and compared among the studied patients by the type of technology used using the Chi-square test. Chi-square Fischer exact tests were used as appropriate to compare the awareness and use of new technologies among the studied patients by their studied demographic- and diabetes-related factors. The p-value of <0.05 was considered as the cut-off value for significance. The odds ratio (OR) for the association of the use of new technologies with the studied demographic- and diabetes-related factors were also calculated.

## Results

A total of 452 patients with diabetes from KSA were included in the study analyses. The characteristics of the studied patients are shown in Table 1. The mean age of the participants was 38.2 ± 12.4 years and 45.15% of them were >40 years. About two-thirds of the studied patients were female (66.8%) and the majority were Saudi citizens (91.4%). The educational level of the studied patients was 69.9% university and higher, 21% secondary education, and 9.1% less than secondary education level. Half of the studied patients were employees (49.6%), and 53.1% reported a monthly income of less than 10,000 SR. Of the studied patients, type 1 diabetes was found in 36.9% and type 2 in 38.8%. Diabetes duration of more than 10 years was found in 160 patients, representing 35.4% of the studied sample. HbA1c level was found to be >7.5% in about half of the studied patients with diabetes (48.5%).

**Table 1:** Characteristics of the studied patients with diabetes (n=452). Data are presented by the mean ± SD or by n (%). SD, standard deviation

Characteristics		n=452
Age in years; mean ± SD (range)		38.2 ± 12.4 (18-60)
Age in years	≤ 40	248 (54.9)
	> 40	204 (45.1)
Sex	Male	150 (33.2)
	Female	302 (66.8)
Nationality	Saudi	413 (91.4)
	Non-Saudi	39 (8.6)
Educational level	Less than secondary	41 (9.1)
	Secondary	95 (21.0)
	University and higher	316 (69.9)
Job	Student	80 (17.7)
	Employee	224 (49.6)
	Not employee	148 (32.7)
Income	< 10000 Saudi Riyal	240 (53.1)
	10-20000 Saudi Riyal	176 (38.9)
	> 20000 Saudi Riyal	236 (8.0)
Type of diabetes	Don't know	167 (36.9)
	Type1	130 (38.8)
	Type2	25 (5.5)
	Gestational	130 (28.8)
Duration of diabetes in years	< 1	88 (19.5)
	1-5	125 (27.7)
	6-10	79 (17.5)
	> 10	160 (35.4)
Hemoglobin A1c level	Don't know	128 (28.3)
	< 6.5	56 (12.5)
	6.5-7.5	105 (23.2)
	> 7.5	163 (36.1)

**Figure 1:**
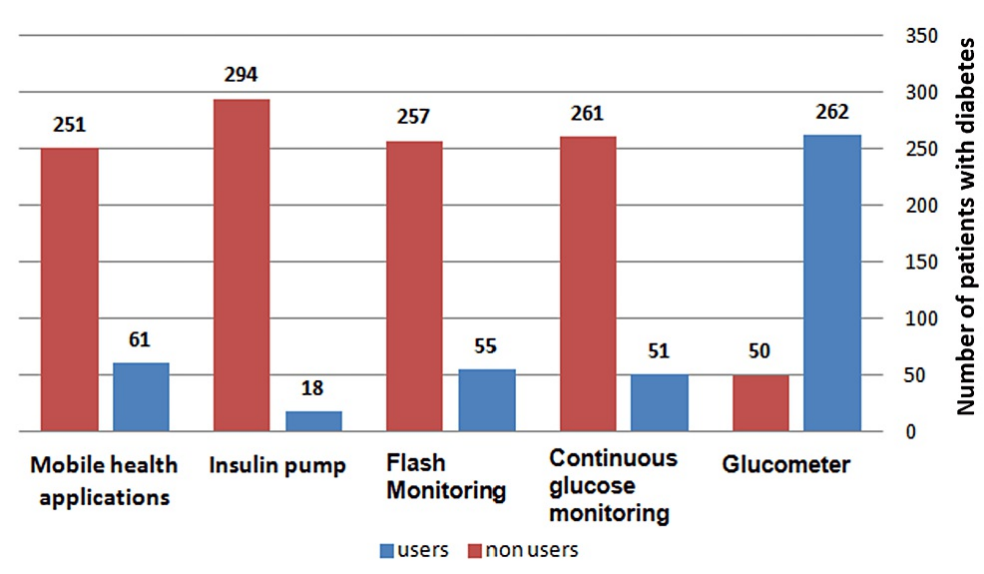
Frequency of use of glucometers and new technology applications among the aware patients with diabetes studied.

Regarding the awareness of new technologies, we found that the majority of participants were aware of it: 312 patients out of the 452 studied patients (69%). The remaining 140 patients (31%) were not aware. The prevalence rates of the use of new technologies in this present study for mobile health applications, flash monitoring, blood glucose monitoring, and insulin pumps among the studied patients with diabetes were 13.5%, 12.2%, 11.3%, and 4%, respectively. Among the studied subjects, 355 reported the use of a glucometer (78.5%). With exception of glucometer use, there were great discrepancies between the awareness by the studied patients of the new technologies and their actual use (Figure 1).

The main cause of not using technologies was their high cost, reported by 53.2% of the respondents (n = 231). Non-availability of a technology and difficulty in its understanding and use was reported by 20.3% and 21.2%, respectively. Lack of advice by treating doctors and fear or lack of trust was reported by 3% and 2.2% of respondents, respectively. Furthermore, the main sources of knowledge were doctors, friends, and social media. Television (TV) played a minor role as a source of knowledge, while newspapers appeared to have no role in acquiring knowledge about new technology in managing diabetes.

For mobile health applications, the most commonly used function among the studied subjects (n = 61) was blood glucose monitoring (93.4%) followed by physical activity programs (44.3%) and nutritional programs (39.3%). Finally, the use of mobile health applications for insulin level determination was reported by only 3.3% of the studied subjects.

Table 2 shows the awareness of the studied patients about new technologies by their characteristics. Significant higher rates of awareness were found among patients aged ≤ 40 years (83.4%) and among students (78.7%). Although not significant, the awareness was also high among male patients (72%), secondary and highly educated patients, and those who reported high monthly family income with an awareness rate of 74.7%, 69.3%, and 80.6%, respectively. For the studied diabetes factors, the rate of awareness was significantly higher among patients with type 1 diabetes (80.8%), and among those patients with HbA1c < 6.5 (80.4%), and HbA1c between 6.5 and 7.5 (83.8%). Although not significant, the rate of awareness was higher among patients with diabetes duration between 6 and 10 years (74.7%). 

**Table 2:** Awareness of the studied patients about new technologies by their characteristics (n=452). *A p-value less than 0.05 is considered statistically significant

Characteristics	Subgroups	Aware (n=312)	Not aware (n=140)	p-value*
Age in years	≤ 40	182 (83.4)	66 (26.6)	0.001
	> 40	130 (63.7)	74 (36.3)	
Sex	Male	108 (72.0)	42 (28.0)	0.35
	Female	204 (67.5)	98 (32.5)	
Educational level	Less than secondary	22 (53.7)	19 (46.3)	0.10
	Secondary	61 (74.7)	24 (25.3)	
	University and higher	219 (69.3)	97 (30.7)	
Job	Student	63 (78.7)	17 (21.3)	0.02
	Employee	147 (65.6)	77 (34.4)	
	Not employee	101 (68.2)	47 (31.8)	
Income	< 10000 Saudi Riyal	165 (68.7)	75 (31.3)	0.26
	10-20000 Saudi Riyal	118 (67.0)	58 (33.0)	
	> 20000 Saudi Riyal	29 (80.6)	7 (19.4)	
Type of diabetes	Don't know	73 (56.2)	57 (43.8)	0.0001
	Type1	135 (80.8)	32 (19.2)	
	Type2	85 (65.4)	45 (34.6)	
	Gestational	19 (76.0)	6 (24.0)	
Duration of diabetes in years	< 1	47 (53.4)	41 (46.6)	0.11
	1-5	91 (72.8)	34 (27.2)	
	6-10	59 (74.7)	20 (25.3)	
	> 10	115 (71.9)	45 (28.1)	
Hemoglobin A1c level	Don't know	63 (49.2)	65 (50.8)	0.0001
	< 6.5	45 (80.4)	11 (19.6)	
	6.5-7.5	88 (83.8)	17 (16.2)	
	> 7.5	116 (71.2)	47 (28.8)	

Table 3 presents the satisfaction with the use of different new technologies among the studied patients with diabetes. There were statistically significant differences for all studied satisfaction items according to the type of technology used. Higher rates of satisfaction for all studied items were related to those patients reporting the use of mobile health applications, FGM, and continuous glucose monitoring; the lowest rate was among those patients using a glucometer. Those using an insulin pump reported a high rate of satisfaction for only the first studied satisfaction items. 

**Table 3:** Satisfaction of the use of different new technologies among the studied patients with diabetes. CGM, continuous glucose monitoring; FGM, flash glucose monitoring *A p-value less than 0.05 is considered statistically significant

Satisfaction items	Agree n (%)					p-value^*^
	Glucometer (n=355)	CGM (n=51)	FGM (n=55)	Insulin pump (n=18)	Mobile health applications (n=61)	
I feel generally fine with my diabetes treatment	40 (11.3)	43 (84.3)	48 (87.3)	13 (72.2)	57 (93.4)	0.0001
Easy and comfortable treatment method	39 (10.9)	42 (82.3)	49 (89.1)	12 (66.7)	54 (88.5)	0.0001
I feel confident dealing with my diabetes	38 (10.5)	40 (78.4)	46 (83.6)	12 (66.7)	54 (88.5)	0.0001
I am not worried about low blood sugar	28 (7.9)	29 (56.9)	32 (58.1)	7 (38.9)	43 (70.5)	0.0001
I am not worried about high blood sugar	25 (7.0)	26 (50.9)	29 (52.7)	8 (44.4)	40 (65.6)	0.0001

Table 4 shows the use of CGM and FGM among the studied patients with diabetes according to their demographic- and diabetes-related factors. Statistically significant differences were detected among the studied patients according to all studied demographic factors, with the exception of income, for both techniques. For CGM, a higher rate of use was found among patients aged ≤40 years (16.1%), female patients (11.9%), highly educated patients (13.3%), and students (22.5%). For the studied diabetes factors, a higher rate of use was found among patients with type 1 diabetes (23.4%), with an OR of 6.3 for those patients. Also, the use was higher among patients with a duration of diabetes of more than 10 years (15%). Significantly increased use of CGM was also found among patients with HbA1c level <6.5 (17.9%) and among those with HbA1c levels between 6.5 and 7.5 13 (18.1%). For FGM, the higher rate of use was among patients aged ≤40 years (18.5%), highly educated patients (15.2%), and students (26.3%). A significantly higher rate of use was found among patients with type 1 diabetes (28.1%) and among those with HbA1c level between 6.5 and 7.5 (21%), with a higher probability of use as indicated by the calculated ORs. 

**Table 4:** Use of CGM and FGM among the studied patients by their demographic- and diabetes-related factors. *A p-value less than 0.05 is considered statistically significant CGM, continuous glucose monitoring; FGM, flash glucose monitoring; OR, odds ratio

Device name		CGM				FGM			
Characteristics		Use (n=51)	Not use (n=401)	OR	p-value^*^	Use (n=55)	Not use (n=397)	OR	p-value^*^
Age in years	≤ 40	40 (16.1)	208 (83.9)	1.00	0.0001	46 (18.5)	102 (81.5)	1.00	0.0001
	> 40	11 (5.4)	193 (94.6)	0.30		9 (4.4)	195 (95.6)	0.10	
Sex	Male	15 (10.0)	135 (90.0)	1.00	0.002	17 (11.3)	133 (88.7)	1.00	0.01
	Female	36 (11.9)	266 (88.1)	1.20		38 (12.6)	264 (87.4)	1.15	
Educational level	Less than secondary	1 (2.4)	40 (97.6)	1.00	0.04	0 (0.0)	41 (100.0)	1.00	0.01
	Secondary	8 (8.4)	87 (91.6)	3.70		7 (7.4)	88 (92.6)	-	
	University and higher	42 (13.3)	274 (86.7)	6.10		48 (15.2)	268 (84.8)	-	
Job	Student	18 (22.5)	62 (77.5)	100	0.02	21 (26.3)	59 (73.7)	1.00	0.0001
	Employee	19 (8.5)	205 (91.5)	0.30		14 (6.3)	210 (93.7)	0.20	
	Not employee	14 (9.5)	134 (90.5)	0.36		20 (13.5)	128 (86.5)	0.45	
Income	< 10000 Saudi Riyal	26 (10.8)	214 (89.2)	1.00	0.12	29 (12.1)	211 (87.9)	1.00	0.09
	10-20000 Saudi Riyal	18 (10.2)	158 (89.8)	0.95		18 (10.2)	158 (89.8)	0.83	
	> 20000 Saudi Riyal	7 (19.4)	29 (80.6)	2.00		8 (22.2)	28 (77.8)	2.10	
Type of diabetes	Don't know	6 (4.6)	124 (95.4)	1.00	0.0001	3 (2.3)	127 (97.7)	1.00	0.0001
	Type1	39 (23.4)	128 (76.6)	6.30		47 (28.1)	120 (71.9)	16.5	
	Type2	5 (3.8)	125 (96.2)	0.95		3 (2.3)	127 (97.7)	1.00	
	Gestational	1 (4.0)	24 (96.0)	0.85		2 (8.0)	23 (92.0)	3.70	
Duration of diabetes in years	< 1	5 (5.7)	83 (94.3)	1.00	0.16	5 (5.7)	83 (94.3)	1.00	0.12
	1-5	14 (11.2)	111 (88.8)	2.10		14 (11.2)	111 (88.8)	2.10	
	6-10	8 (10.1)	71 (89.9)	1.90		12 (15.2)	67 (84.8)	3.00	
	> 10	24 (15.0)	136 (85.0)	2.90		24 (15.0)	136 (85.0)	2.90	
Hemoglobin A1c level	Don't know	3 (2.3)	125 (97.7)	1.00	0.0001	3 (2.3)	125 (97.7)	1.00	0.0001
	< 6.5	10 (17.9)	46 (92.1)	9.00		8 (14.3)	48 (85.7)	6.90	
	6.5-7.5	19 (18.1)	86 (81.9)	9.20		22 (21.0)	83 (79.0)	11.0	
	> 7.5	19 (11.6)	144 (88.4)	5.50		22 (13.5)	141 (86.5)	6.50	

Table 5 shows the use of insulin pumps and mobile health applications among the studied patients with diabetes by their demographic- and diabetes-related factors. Except for age, the use of insulin pumps did not show statistically significant differences in the studied demographic factors. However, a higher rate of use of insulin pumps was found among patients aged ≤40 years (6.5%), males (5.3%) 14 and students (8.8%), and among those with high income (8.3%). For the studied diabetes factors, a significantly higher rate of insulin pump usage was found among patients with type 1 diabetes (10.2%), duration of diabetes of more than 10 years (8.1%), and among those patients with HbA1c levels between 6.5 and 7.5 (9.5%). A significantly higher rate of use of mobile health applications was found among patients aged ≤40 years (21%) and among students (26.3%). Although not significant, the use of mobile health applications was also higher among male patients (17.3%), highly educated patients (15.2%), and among those patients reporting high-income levels (19.4%). For the studied diabetes factors, a significantly higher rate of mobile health application usage was found among patients with type 1 diabetes (28.1%), and 19.6% and 20% among those patients with HbA1c levels of <6.5 and between 6.5 and 7.5, respectively.

**Table 5:** Use of insulin pump and mobile health applications among the studied patients by their demographic- and diabetes-related factors. *A p-value less than 0.05 is considered statistically significant OR, odds ratio

Device name		Insulin pump				Mobile health applications			
Characteristics		Use (n=18)	Not in use (n=434)	OR	p-value^*^	Use (n=61)	Not in use (n=391)	OR	p-value*
Age in years	≤ 40	16 (6.5)	232 (93.5)	1.00	0.0001	52 (21.0)	196 (79.0)	1.00	0.0001
	> 40	2 (1.0)	203 (99.0)	0.15		9 (4.4)	195 (95.6)	0.17	
Sex	Male	8 (5.3)	142 (94.7)	1.00	0.14	26 (17.3)	124 (82.7)	1.00	0.15
	Female	10 (3.3)	292 (96.7)	0.60		35 (11.6)	267 (88.4)	0.60	
Educational level	Less than secondary	0 (0.0)	41 (100.0)	1.00	0.09	0 (0.0)	41 (100.0)	1.00	0.12
	Secondary	3 (3.2)	92 (96.8)	-		13 (13.7)	82 (82.3)	-	
	University and higher	15 (4.7)	301 (95.3)	-		48 (15.2)	268 (84.8)	-	
Job	Student	7 (8.8)	73 (91.2)	1.00	0.06	21 (26.3)	59 (73.7)	1.00	0.0001
	Employee	6 (2.7)	218 (97.3)	0.30		22 (9.8)	202 (90.2)	0.30	
	Not employee	5 (3.4)	143 (96.6)	0.35		18 (12.2)	130 (87.8)	0.40	
Income	< 10000 Saudi Riyal	11 (4.6)	229 (95.4)	1.00	0.09	32 (13.3)	208 (86.7)	1.00	0.27
	10-20000 Saudi Riyal	4 (2.3)	172 (97.7)	0.48		22 (12.5)	154 (87.5)	0.95	
	> 20000 Saudi Riyal	3 (8.3)	33 (91.7)	1.90		7 (19.4)	29 (80.6)	1.60	
Type of diabetes	Don't know	0 (0.0)	130 (100.0)	1.00	0.0001	2 (1.5)	128 (98.5)	1.00	0.0001
	Type1	17 (10.2)	150 (89.8)	-		47 (28.1)	120 (71.9)	25.0	
	Type2	1 (0.8)	129 (99.2)	-		12 (9.2)	118 (90.8)	6.50	
	Gestational	0 (0.0)	25 (100.0)	-		0 (0.0)	25 (1.00)	-	
Duration of diabetes in years	< 1	0 (0.0)	88 (100.0)	1.00	0.0001	7 (8.0)	81 (92.0)	1.00	0.60
	1-5	1 (0.8)	124 (99.2)	-		18 (14.4)	107 85.6)	1.95	
	6-10	4 (5.1)	75 (94.9)	-		12 (15.2)	67 (84.8)	2.10	
	> 10	13 (8.1)	147 (91.9)	-		24 (15.0)	136 (85.0)	2.05	
Hemoglobin A1c level	Don't know	1 (0.8)	127 (99.2)	1.00	0.0001	1 (0.8)	127 (99.2)	1.00	0.0001
	< 6.5	2 (3.6)	54 (96.4)	4.70		11 (19.6)	45 (80.4)	31.0	
	6.5-7.5	10 (9.5)	95 (90.5)	13.4		21 (20.0)	84 (80.0)	32.0	
	> 7.5	5 (3.2)	158 (96.8)	4.00		28 (17.2)	135 (82.8)	26.0	

## Discussion

Technologies used in managing diabetes have advanced in recent years with the introduction of different types of devices. In this cross-sectional study, we aimed to measure the awareness of the modern technologies used in managing diabetes among patients with diabetes in Saudi Arabia and their satisfaction while using them; we found that out of 452 patients with diabetes, 312 (69%) of the sample were aware of these technologies. There was a significantly higher rate of awareness among patients aged ≤40 years (83.4%) and among students (78.7%). For the studied diabetes factors, the rate of awareness was significantly higher among patients with type 1 diabetes (80.8%), and among those patients with HbA1c of <6.5 (80.4%), and HbA1c between 6.5 and 7.5 (83.8%). So, younger patients with better control showed better awareness, but it could be the other way that the younger patients who showed better awareness had better control as a result.

The prevalence of the use of modern technology among the studied patients with diabetes was higher for mobile health applications (13.5%) followed by FGM (12.2%) and CGM (11.3%). A possible explanation for this trend is the cost and ease of accessibility. Also, our study showed that the use of glucometers was more widespread than newer technologies among the aware patients with diabetes studied (Figure 1).

We found that the main sources of knowledge about the new technologies in managing diabetes were doctors, friends and social media, while the TV played a minor role as a source of knowledge. In the present study, the causes of not using these technologies among the studied patients were high cost, as reported by 53.2% of the respondents (n = 231), non-availability of such technologies (20.3%), difficulty in understanding and use (21.2%), lack of advice from treating doctors (3%), and fear and lack of trust (2.2%). Previous studies [12-14] reported that the most common cause of non-use or discontinuing device use was cost-related. Another study showed reduced usage of mobile applications due to insufficient doctor's advice on the use of apps for diabetes management [15].

While comparing the use of new technology among the patients with diabetes studied by their demographic and diabetes-related factors we found that the use of new technologies for diabetes management was significantly associated with age ≤ 40 years, type 1 diabetes, and among those patients with HbA1c level between 6.5 and 7.5. There was a higher rate of use among students for all new technologies except for the insulin pump. Also, higher education was significantly associated with the use of CGM and FGM. The one technology that was associated significantly with a duration of diabetes of more than 10 years was the insulin pump. A study by Rafiullah and David [16] in Saudi Arabia showed that the use of health applications was slightly higher (45.91%) among young patients. Also, high educational levels seemed to affect the extent of using health applications.

Regarding mobile health applications, the majority of the patients with diabetes studied used them for blood glucose monitoring (93.4%), physical activity programs (44.3%) and nutritional programs (39.3%), and a few of them used applications for insulin dose determination (3.3%). Similar to our results, Rafiullah and David [16] found that the most common use of mobile health applications was for blood glucose measurement (21.97%) and exercise (18.38%).

Some studies [17-18] noted that greater improvement in blood glucose in patients using mobile platforms or CGM indicated that technologies can enhance diabetes care.

A study by Vaala et al. [19] noted that many different technologies offered professional information about diabetic management that aimed to increase awareness and effectiveness among patients, particularly young patients, such as social media, websites, diabetic applications, text messaging, and pump/glucometer software.

Comparing new technologies to a glucometer, which is the traditional method used in diabetes management, a higher rate of use was found among patients with type 1 (91%) and type 2 (87.7%) DM with ORs of 7.4 and 5.2, respectively, among those patients. Also, we found that the use of glucometers was higher among patients with a duration of diabetes of more than five years and those with higher HbA1c levels.

Patient satisfaction plays an important role in the adherence and success of diabetes management. As this study measured patient satisfaction with modern devices and the traditional device, we found the higher rate of satisfaction for all studied items was related to the patients who use mobile health applications, FGM, and CGM. The lowest rate of satisfaction was found among patients who reported using a glucometer alone (Table 3).

Our findings were supported by a number of studies. A recent systematic review conducted among Saudi patients who use mHealth applications showed a positive effect on their health-related behaviors and outcomes, with a higher rate of satisfaction in comparison to traditional care [14]. A meta-analysis attributed those results to self-management skills such as symptom awareness, monitoring, and management, which were all facilitated by mHealth applications [20]. Regarding FGM, Al Hayek's study [21], carried out in Saudi Arabia for patients with type 1 diabetes, found that the use of FGM increases the frequency of self-testing, thus helping to reduce the frequency of hypoglycemia and HbA1c level and to improve the quality of life compared to traditional testing methods. A similar result was reported in another Saudi study for patients with type 2 diabetes [22].

According to the American Diabetes Association (ADA), CGM systems have been increasing in popularity and comfort vs. the standard glucometer [2]. Previous studies [10, 23-25] showed that CGM helps in increasing treatment satisfaction and enhances ease of diabetes care, self-management and psychosocial outcome, and improves glycemic control for patients with type 1 and type 2 diabetes.

In the current study, patients using an insulin pump reported a high rate of satisfaction for only three studied satisfaction items: “I feel generally fine with my diabetes treatment,” “Easy and comfortable treatment method,” and “I feel confident dealing with my diabetes” (Table 3). Previous studies [6, 26] comparing the insulin pump with traditional treatment methods found an increase in treatment satisfaction with the insulin pump among the studied population.

Our study is the first conducted in Saudi Arabia that discusses multiple new technologies used in the management of DM. However, it has some limitations, as the collection of data was via an online survey, which could have some recall bias and issues of limited sampling.

## Conclusions

In conclusion, with the current rapid development of modern technologies in managing diabetes, our findings revealed that the use of these technologies is still limited despite the majority of the participants being aware of them. The main barrier to the non-use of new technologies was primarily their high cost. According to the data, the use of new technologies was more common among certain demographic- and diabetes-related factors, such as the age, type, and duration of diabetes. However, modern technologies have a higher rate of satisfaction in comparison to conventional methods. As the government continues to support patients with diabetes who use traditional methods by distributing glucometers for free to every patient with diabetes in their facilities, it also started providing modern diabetes technologies, such as FGM and insulin pumps, mostly for type 1 patients. Thus, we recommend greater government support and launching financial support programs for patients with diabetes to help them cover the cost of these technologies. We also recommend physicians to encourage their patients in using mobile health applications, as it provides better satisfaction and is considered of a lower cost. This study had some limitations such as being conducted as an online survey; therefore, more research is needed to corroborate these findings in clinical settings and with a larger sample of participants from all of Saudi Arabia's varied communities to obtain more reliable and focused results.

## Appendices

Research questionnaire

This questionnaire is for patients with diabetes in Saudi Arabia. It aims to measure patients' perspectives of the new technologies that are used to manage diabetes and their satisfaction regarding these technologies.

• Participation is optional.

• Your information will be strictly confidential as soon as you complete the questionnaire.

_____________________________

Demographic information (personal information)

Age: ..................

Gender:

1. Female.

2. Male.

Nationality:

1. Saudi.

2. Non-Saudi.

Educational level:

1. Did not receive the traditional education.

2. Primary/intermediate education.

3. High-school education.

4. University and above education.

Occupation:

1.   Student. 

2.   Employed.

3.   Unemployed.

4.   Student or worker in the health field.

Income Status:

1. High income (>20,000/month).

2. Intermediate income (10,000-20,000/month).

3. Low income (<10.000/month).

Residence:

1.   Madinah Al-Munawarah.

2.   Makkah Al-Mukaramah.

3.   Riyadh.

4.   Jeddah.

5.   Taif.

6.     Dammam.

7.     Others.

Health Information

Which type of diabetes do you have?

1. Diabetes mellitus - Type 1

2. Diabetes mellitus - Type 2

3. Gestational diabetes.

4. I do not know.

If you have diabetes mellitus - Type 2, do you use injecting insulin for treatment?

1.   Yes.

2.   No.

When were you diagnosed with diabetes?

1. Less than a year.

2. 1-5 years.

3. 6-10 years.

4. More than 10 years.

Do you know your hemoglobin A1c test (HbA1c)?

1. Yes

2. No

What is your hemoglobin A1c level?

1. Less than 6.5%.

2. 6.5-7.5%.

3. 7.6-8.5%.

4. More than 8.5%.

Glucometer

It is a device used to monitor the level of glucose in the blood using a drop of blood taken by a finger prick, placed on the device strip, read by the meter, and the result of the level of sugar in the blood was taken.

-------------------------------------------------------------------------------------

Do you use glucometer?

1.     Yes.

2.     No.

How did you first hear about glucometer?

1. Responsible doctor.

2. TV.

3. Magazine/Newspaper.

4. Social media.

5. A friend/relative/associate.

6. Others: ……

Do you advise/recommend glucometer to a friend or colleague?

1. Definitely not.

2. Probably not.

3. Not sure.

4. Probably.

5. Definitely

How long have you used glucometer?

1. Less than a month.

2. 1 to 6 months.

3. 6 months to a year.

4. More than a year.

Are you still using glucometer until now?

1. Yes.

2. No.

Did you get a glucometer freely from the hospital or health center, or did you buy it at your own expense?

1. Yes, I got it from the hospital or health center.

2. No, I bought it at my own expense.

Do you get the glucometer strips regularly and freely from the hospital or health center, or do you buy them at your own expense?

1. Yes, I got it from the hospital or health center.

2. No, I bought it at my own expense.

New technologies used in diabetes management

How well do you know about the new technologies used to manage diabetes, such as [continuous glucose monitoring (CGM), insulin pump, mobile applications, flash glucose monitoring (FGM)]?

1. Never heard of them.

2. I am aware but have never used them.

3. I use them sometimes.

4. I use them regularly.

Why have you not used any of the new technologies? ( you can choose more than one option)

1. Cost.

2. Complicated and difficult to use.

3. Unavailable.

4. Others: …..

If you are willing to use any of these new technologies in the future, which one will be used? ( you can choose more than one option )

1. CGM.

2. Insulin pump.

3. Mobile phone applications.

4. FGM.

5. Nothing.

Continuous glucose monitoring

It is a  device used to measure glucose levels every few minutes using a sensor (needle), which is inserted under the skin (usually in the abdominal wall or arm).

-----------------------------------------------------------------------------------------

Do you use/have you previously used continuous glucose monitoring?

1.   Yes.

2.   No.

How did you first hear about CGM?

1. Responsible doctor.

2. TV.

3. Magazine/Newspaper.

4. Social media.

5. A friend/relative/associate.

6. Others: ……

Do you advise/recommend CGM to a friend or colleague?

1. Definitely not.

2. Probably not.

3. Not sure.

4. Probably.

5. Definitely

How long have you used CGM?

1. Less than a month.

2. 1 to 6 months.

3. 6 months to a year.

4. More than a year.

Are you still using CGM until now?

1. Yes.

2. No.

Flash glucose monitoring

It is a device used to monitor glucose levels using flash technology in which glucose levels are measured in the interstitial fluid under the skin by placing a sensor on the arm skin, and the readings are obtained by scanning with the specified device or by some mobile devices without the need for a prick.

--------------------------------------------------------------------------------------

Do you use/have you previously used FGM?

1.   Yes.

2.   No.

How did you first hear about FGM?

1. Responsible doctor.

2. TV.

3. Magazine/Newspaper.

4. Social media.

5. A friend/relative/associate.

6. Others: ……

Do you advise/recommend FGM to a friend or colleague?

1. Definitely not.

2. Probably not.

3. Not sure.

4. Probably.

5. Definitely

How long have you used FGM?

5. Less than a month.

6. 1 to 6 months.

7. 6 months to a year.

8. More than a year.

Are you still using FGM until now?

1. Yes.

2. No.

Insulin pump

It is a small pumping device that contains an insulin reservoir and a tube for delivering insulin which ends with a thin plastic needle that is inserted under the skin; to deliver certain amounts of insulin found in the reservoir to the body automatically.

---------------------------------------------------------------------------------

Do you use/have you previously used insulin pump?

1.   Yes.

2.   No.

How did you first hear about insulin pump?

1.   Responsible doctor.

2.   TV.

3.   Magazine/Newspaper.

4.   Social media.

5.   A friend/relative/associate.

6.   Others: ……

Do you advise/recommend insulin pump to a friend or colleague?

1. Definitely not.

2. Probably not.

3. Not sure.

4. Probably.

5. Definitely

How long have you used insulin pump?

1. Less than a month.

2. 1 to 6 months.

3. 6 months to a year.

4. More than a year.

Are you still using insulin pump until now?

1. Yes.

2. No.

Mobile phone applications

These are useful applications and programs for patients with diabetes which help them to monitor blood sugar levels, remind them of medication time, or help them to calculate and choose the foods they eat to maintain their calories count, so they can manage their health better.

---------------------------------------------------------------------------------

Do you use/have you previously used Mobile phone application for managing diabetes?

1.   Yes

2.  No

For which purpose do you use mobile phone applications for managing diabetes?

1.   Nutrition applications.

2.   Physical activity applications.

3.   Glucose monitoring applications.

4.   Insulin measurement applications.

5.   Insulin delivery applications.

How did you first hear about mobile phone applications for managing diabetes?

1. Responsible doctor.

2. TV.

3. Magazine/Newspaper.

4. Social media.

5. A friend/relative/associate.

6. Others: ……

Do you advise/recommend mobile phone applications for managing diabetes to a friend or colleague?

1. Definitely not.

2. Probably not.

3. Not sure.

4. Probably.

5. Definitely

How long have you used mobile phone applications for managing diabetes?

1. Less than a month.

2. 1 to 6 months.

3. 6 months to a year.

4. More than a year.

Are you still using mobile phone applications for managing diabetes until now?

1. Yes.

2. No.

**Table 6:** Patient's satisfaction measurement. Patient satisfaction was measured for each device.

Items to measure the patient's satisfaction	Strongly agree	Agree	Neutral	Disagree	Strongly disagree
I am fully satisfied with my diabetes treatment.					
My treatment method is easy and convenient.					
I feel confident dealing with diabetes.					
I have no worries about hypoglycemia					
I have no worries about hyperglycemia					
